# Effects on temporomandibular disorder in the treatment of tension-type headache with acupuncture and therapeutic exercises. A secondary analysis from a randomized controlled trial

**DOI:** 10.1177/02692155241229282

**Published:** 2024-02-02

**Authors:** Joerg Schiller, Alina Büttner, Daniel Niederer, Andrea Bökel, Christoph Korallus, Christian Sturm, Lutz Vogt, Christoph Gutenbrunner, Matthias Karst, Matthias Fink, Christoph Egen

**Affiliations:** 1 Department of Rehabilitation and Sport Medicine, 9177 Hannover Medical School, Hannover, Germany; 2 Department of Sports Medicine and Exercise Physiology, Institute of Occupational, Social and Environmental Medicine, 9173 Goethe University Frankfurt, Frankfurt am Main, Germany; 3 Department of Sports Medicine and Exercise Physiology, 9173 Goethe University Frankfurt, Frankfurt am Main, Germany; 4 Department of Anesthesiology and Intensive Care Medicine, 9177 Hannover Medical School, Hannover, Germany

**Keywords:** Tension-type headache, temporomandibular (joint) Disorder, combination therapy, acupuncture, therapeutic exercise

## Abstract

**Objectives:**

To examine the effects of acupuncture and therapeutic exercise alone and in combination on temporomandibular joint symptoms in tension-type headache and to evaluate the potential interaction of existing temporomandibular dysfunction on the success of headache treatment.

**Design:**

Pre-planned secondary analysis of a randomized controlled, non-blinded trial.

**Setting:**

Outpatient clinic of a German university hospital.

**Subjects:**

Ninety-six Participants with frequent episodic or chronic tension-type headache were randomized to one of four treatment groups.

**Interventions:**

Six weeks of acupuncture or therapeutic exercise either as monotherapies or in combination, or usual care. Follow-up at 3 and 6 months.

**Main measures:**

Subjective temporomandibular dysfunction symptoms were measured using the Functional Questionnaire Masticatory Organ, and the influence of this sum score and objective initial dental examination on the efficacy of headache treatment interventions was analyzed.

**Results:**

Temporomandibular dysfunction score improved in all intervention groups at 3-month follow-up (usual care: 0.05 [SD 1.435]; acupuncture: −5 [SD 1.436]; therapeutic exercise: −4 [SD 1.798]; combination: −3 [SD 1.504]; *P* = 0.03). After 6 months, only acupuncture (−6 [SD 1.736]) showed a significant improvement compared to the usual care group (*P* < 0.01). Subjective temporomandibular dysfunction symptoms had no overall influence on headache treatment.

**Conclusions:**

Only acupuncture had long-lasting positive effects on the symptoms of temporomandibular dysfunction. Significant dental findings seem to inhibit the efficacy of acupuncture for tension-type headache.

## Introduction

In addition to drug therapy strategies, frequent tension-type headache can be treated with non-drug methods such as acupuncture and physiotherapeutic exercise.^1–3^ Acupuncture used alone or in combination with physiotherapeutic exercise reduced pain significantly^4^ and had positive effects on depression, anxiety, and quality of life.^5^ Acupuncture can induce analgesic effects by activating various pain inhibitory control systems, and therapeutic exercise may have an additional regulatory effect on the autonomic nervous system and can be associated with positive neuroplastic changes.^6,7^ Therefore, an additive effect on chronic pain conditions from combined acupuncture and therapeutic exercise applications can be assumed.

Based on the anatomical and functional relationship and the similarities of symptomatology a close relationship between the head and the temporomandibular joint can be suspected.^8^ A temporomandibular disorder manifests itself clinically locally and regionally in the head and neck area.^9^ From the perspective of dentistry, various occlusal disorders are etiologically linked to the development of temporomandibular dysfunction.^10^ Both diseases affect the same nociceptive system and utilize trigeminocervical structures that are involved in central pain modulation.^11^ Temporomandibular dysfunction is discussed as a possible trigger factor for the manifestation of tension-type headache, as well as a predictor of it becoming chronic.^12^ A coincidence of tension headache and temporomandibular dysfunction is known from the literature and a multi-professional approach is recommended.^8^ Numerous studies have shown that treatment of temporomandibular dysfunction has an effect on headache.^11^ However, we are not aware of any work that has examined the influence of multimodal headache treatment on additional temporomandibular joint complaints in comparison to monotherapy with a control group. The purpose of this study is to examine the effects of tension-type headache treatments on subjective temporomandibular disorder in terms of synergistic effects. Furthermore, it was investigated to which extent temporomandibular dysfunction represents a potential confounder for successful treatment of tension-type headache.

## Methods

This study is a randomized-controlled, prospective, non-blinded clinical trial with four treatment arms of equal sizes: therapeutic exercise and acupuncture each as monotherapy, the two in combination, and usual care for the control group. The secondary outcome parameters, which were analyzed in this paper, focus on impairments associated with the jaw joint and are determined using dental examinations and a questionnaire for the subjective assessment of the function of the masticatory system. These secondary outcomes were further investigated by exploratory association analysis with some of the primary outcome measures. The methods have been published in the study protocol^13^ and the primary outcome parameters (pain intensity and frequency of headache) have already been published.^4^ The ethics committee of the Hannover Medical School approved the study (No. 7751_BO_S_2018). It is registered in the German Clinical Trials Register under the number DRKS000167231.

All examinations and interventions were performed in the outpatient Department of Rehabilitation Medicine at Hannover Medical School. We included persons aged 18 to 65 years who had suffered from frequent episodic tension-type headache (1–14 days/month) or chronic tension-type headache (>14 days/month) for more than 6 months, with at least one episode/month for at least 3 months, symptomatology had to meet the diagnostic criteria 2.2 or 2.3 of the *International Classification of Headache Disorders*, 3rd Edition.^14^ After conducting an individual medical history interview, participants who reported pregnancy and breastfeeding, severe physical or psychiatric illnesses, substance abuse, use of headache medication for more than 10 days/month, >1 migraine attack/year or acupuncture or therapeutic exercise treatment for tension headaches within the past 6 months were excluded from the study.

The recruitment of 212 interested participants took place between July 2018 and May 2019 via regional media and pain medicine institutions. Initially, a medical and physical examination was performed, resulting in verification of the diagnosis by specially trained experienced physicians. Subsequently, all participants were informed about the IHS guideline-compliant treatment, the study background and procedure, and the benefits and risks of the planned interventions. Ninety-six participants were enrolled in the study after providing written informed consent. Each person received a study ID and was randomly assigned to one of the four treatment arms by drawing an opaque, sealed envelope containing the treatment arm assignment. The envelopes had been prepared in advance using balanced randomization lists with varying permutation blocks. According to the assignment, each study participant received an individual therapy plan. Furthermore, an appointment was scheduled for a dental examination to assess the clinical functional status according to the German Society of Craniomandibular Function and Disorders.^15^ Regardless of the temporomandibular findings, participants did not receive any additional temporomandibular dysfunction-specific counseling or treatment. Prophylactic or abortive medication was not allowed to change during the study period and was stable for a period of 3 months prior to the start of the study; the use of acute pain medication had to be documented. Study participants could withdraw their participation or terminate therapy at any time without giving a reason. To ensure study compliance, regular personal visits were conducted with study staff members.

For each participant, 12 treatments took place within 6 weeks. The frequency was three times/week in the first two weeks, two treatments per week in the following two weeks, and one treatment per week in the last two weeks. The intervention designs were identical in frequency to counteract systematic bias. Duration and setting correspond to patient expectations and national standards. Participants in the usual care group received no additional therapies, comparable to a waiting group. They were encouraged to continue their guideline-compliant^1^ symptomatic treatment with analgesics for acute headache attacks, taken on fewer than 10 days/month, as usual (e.g. nonsteroidal anti-inflammatory drugs (NSAIDs), paracetamol, and their combination with caffeine). In chronic tension-type headache, pharmacological (e.g. amitriptyline, mirtazapine, and venlafaxine) and non-pharmacological prophylactic treatment strategies (e.g. electromyographic (EMG) biofeedback, cognitive-behavioral therapy, and relaxation techniques) should have also been continued according to the guideline recommendations outlined above. In order to encourage compliance of the usual care group participants, free acupuncture or therapeutic exercise was offered at the end of the observation phase.

Participants in the acupuncture group received acupuncture treatments without additional treatment measures such as moxibustion or cupping. The acupuncture was applied according to the principles of traditional Chinese medicine (TCM), current scientific literature^2^ and in collaboration with experts from the Chinese Academy of Chinese Medical Science (CACMS, Beijing) and experienced instructors in acupuncture/TCM from Germany. There is enormous diversity in the use of acupuncture treatment in terms of the points used, the number of points, the duration of the sessions, the frequency of treatment, and the duration of the treatment programs and clear guidelines have not been scientifically proven.^16^ Therefore, in a balanced discussion among a peer group of acupuncturists, we decided to start the treatment sessions with a high frequency to initially “boost” the effectiveness of acupuncture and then gradually reduce it to one session per week. Our peer group consisting of TCM experts from CACMS and acupuncturists from the Hannover Medical School recommended this method as a best practice treatment protocol. Sterile disposable acupuncture needles (25–40 mm × 0.25–0.3 mm; Suzhou-Tianxie) were used by an acupuncturist with more than 5 years of professional experience. The needles were manually stimulated after insertion and after 30 min to induce a “deqi*”* sensation. The choice of acupuncture points was semi-standardized: As standard, four points were needled and supplemented by three to five individual points according to the reported pain localization (Table 1).

**Table 1.** Detailed description of the interventions.^7,8^

Treatment: Acupuncture (30 min)			
Selection of acupuncture points	Acupuncture points	Depth (cun)	Duration (min)
Standardized points	Baihui (GV20)	0.5–0.8	30
	Taiyang (EX-HN5)*	0.3–0.5	
	Fengchi (GB20)*	0.8–1.2	
	Hegu (LI4)*	0.5–1.0	
Pain localization (Meridian)			
Front head, forehead, brow edge (Yangming)	Neiting (ST44)*	0.5–0.8	
	Yintang (GV29)*	0.3–0.5	
Side head, temporal (Shaoyang)	Zulinqi (GB41)*	0.3–0.5	
	Waiguan (SJ5)*	0.5–1.0	
Back head, occipital (Taiyang)	Kunlun (BL60)*	0.5–1.0	
	Houxi (SI3)*	0.5–1.0	
Top of the head (Jueyin)	Taichong (LI3)*	0.5–0.8	
	Neiguan (PC6)*	0.5–1.0	
	Sishencong (EX-HN1)**	0.5–0.8	

Treatment: Therapeutic exercise (60 min)			
Method	Intervention		Duration (min)
Cardiovascular training	Bicycle ergometer training***		15
	Rowing-machine***		
	Crosstrainer***		
	Treadmill***		
Strength-endurance training	Pull-down***		10–15
	Butterfly reverse***		
	Row***		
Coordinative training	Postural training***		5–10
Propriozeptiv training	Segmental stabilization of the spine***		5
Training of mobility and flexibility	Specific stretching/relaxation program of the muscles		10
	Active self-mobilization of the spine		

Cun: Traditional Chinese Medicine unit of measurement for locating acupuncture points, width of the distal phalanx of the thumb. GV: governor vessel; EX-HN: extra point head and neck; GB: gallbladder; LI: large intestine; ST: stomach; SJ: San Jao; BL: urinary bladder; SI: small intestine; LI: heart; PC: pericardium; *: bilateral needling; **: quadruple; ***: exercise equipment (Frei medical GmbH and Haider Bioswing GmbH).

The therapeutic exercise (called Medical Training Therapy in Germany) is a physician-prescribed active form of physiotherapy focusing on muscle strength, endurance, and cardiopulmonary capability.^17^ The exercise program was designed and individually dosed by experienced physiotherapists. Active therapy strategies are expressly recommended in the treatment guidelines^1^ and enable those affected to continue this treatment independently even after the study has ended. The main components of the one-hour training program were initial indoor cycling at an intensity of 75% of the previously calculated maximum heart rate. This was followed by strength training on medical training machines with an intensity of 60% to 80% of maximum strength according to the individual constitution as well as postural-proprioceptive training with flexible oscillating rods and unstable oscillating surfaces, flexibility and coordinative training. In order to optimize the mobility of the spine, instructions were given for active self-mobilization of the spine (Table 1). Initially, a 45-min physiotherapy assessment was performed in order to develop an individualized treatment plan. The therapeutic exercise took place in groups of up to four participants and was instructed by a physiotherapist in the department’s gym.

In the combination group, participants first received therapeutic exercise as described and, after a 10-min break, acupuncture treatment. All participants in the therapeutic exercise and combination groups were requested to continue the therapy program they had learned independently and continuously after the end of the 6-week intervention phase. In addition, they received a compact written 15-min self-exercise program that they had to perform three times per week throughout the entire observation period.

In order to assess the improvement of temporomandibular joint-associated complaints in the course of headache treatment, the Functional Questionnaire Masticatory Organ^18^ was used. An optimized function of the jaw masticatory system could be explained by an improvement in regional blood flow, a reduction in muscle tone and regional analgesic therapeutic effects of headache treatment as well as effects on central pain perception and processing. The validated questionnaire consists of 12 items that assess subjective dysfunctions in the area of mandibular mobility and myopathies of the masticatory and mimic muscles. In terms of content, questions ask about complaints when moving the lower jaw, speaking normally, biting, or licking the lips. Other items assess problems with solid food intake, chewing, and eating in large bites. Data on mandibular mobility were collected by reporting difficulties in opening the mouth wide, yawning, laughing, or rinsing out the mouth as usual. In addition, the participants were asked to indicate whether they had sleeping disorders due to the presenting temporomandibular dysfunction symptoms. For each of the 12 questions, the questionnaire contains a 10-point response scale (1 = no limitations at all, 10 = intolerable discomfort), so that 12–120 points can be assigned as a sum score. Over the entire study period of 7 months, participants were examined at fixed time points. After completion of the six-week intervention phase, a follow-up phase was conducted, with two follow-up appointments three (T3) and six (T4) months after the start of therapy.

Pain intensity from tension-type headache was assessed at baseline (T0) and at the follow-up appointments using a numeric rating scale. The frequency of headache was recorded using a headache diary.^4^

A dental examination was performed once at baseline (T0) as opposed to multiple temporomandibular dysfunction scores and headache activity measures. A specially trained dentist performed the objective assessment of temporomandibular dysfunction using an examination form based on the clinical functional status of the German Association for Dental, Oral, and Maxillofacial Medicine. On the one hand, the examination focused on the intraoral triggering factors such as occlusal disorders and missing teeth in the support zone area. On the other hand, the detectable consequences of temporomandibular dysfunction were assessed. Auscultated crepitating and cracking sounds, pain on bilateral palpation, limited mandibular mobility, and the expression of discomfort or pain during the examination were documented. Intraorally, the presence of abrasions/attritions, tongue/cheek impressions, wedge defects, enamel cracks, and gingival recessions were also assessed. The dental examination score consisted of a total of 14 dichotomously assessed (pathological = yes/no) examination parameters. The assessment comprised 12 items with one point each. For two items (“palpation/auscultation of the temporomandibular joint”), the examination findings were differentiated on the right and left sides so that one point could be achieved for each side. These results were used to calculate the dental examination score, which ranged from 0 to 16 points. A higher score indicates more severe objective dental pathologies. In addition, data on dental/medical treatments and functional therapies, occlusal splints and relevant temporomandibular joint disorders were recorded.

The sample size was calculated for the primary outcome parameter average pain intensity from the tension-type headache based on the work of Endres et al.^19^ and resulted (interaction effect size *f* = 0.15, α=0.05, beta = 80% for five measurement time points and four study groups) in a group sample of 24 participants (*n* = 96) with a 20% drop-out rate. After reaching the required sample size, recruitment was closed.

To reveal potential between-groups differences at baseline, T0 values were compared using one-way analyses of variances (ANOVAs) for all interval scaled metric data, and cross-tables with chi-squared tests (when the underlying assumptions were fulfilled, else Fishers’ exact tests) for ordinal and nominal scaled data. The following main analyses were performed for change scores (changes to T0) for T3 and T4 for the temporomandibular dysfunction-score.^2^ Analyses were carried out on an intention-to-treat-basis. Repeated measures ANOVAs (rmANOVAs) were calculated for the main effects (group and time), for the interaction effect (group×time) and for a potential co-variate contribution (group×time×baseline-value; group×time×pericranial tenderness) repeated measures one-way analysis of covariance (rmANCOVAs). All rmANCOVAs were performed following the checking for potential violations of the underlying assumptions for parametric variance analyses (heteroscedasticity, non-linearity, and normal distribution of the residuals). For significant omnibus tests, (adjusted for covariances) post-hoc tests with least square differences were calculated. Associated (adjusted, likewise) data was displayed as means and 95% confidence intervals. Explorative analyses contained afterwards association analyses. First, a method comparison between objective (dental examination score), and self-report impairment resulting of the temporomandibular joint disorders was investigated using Pearson's correlation analyses and Bland–Altman methods comparisons. A potential contribution of temporomandibular dysfunction on acupuncture/therapeutic exercise effects was afterwards investigated by a re-analysis of the outcomes published in 2021.^4^ All analyses were performed using SPSS (Version 24, IBM SPSS, USA). For all significance testing, an alpha-error of 5% was considered as a valid significance cut-off, all *P*-values below were interpreted as significant.

## Results

**Figure 1.**
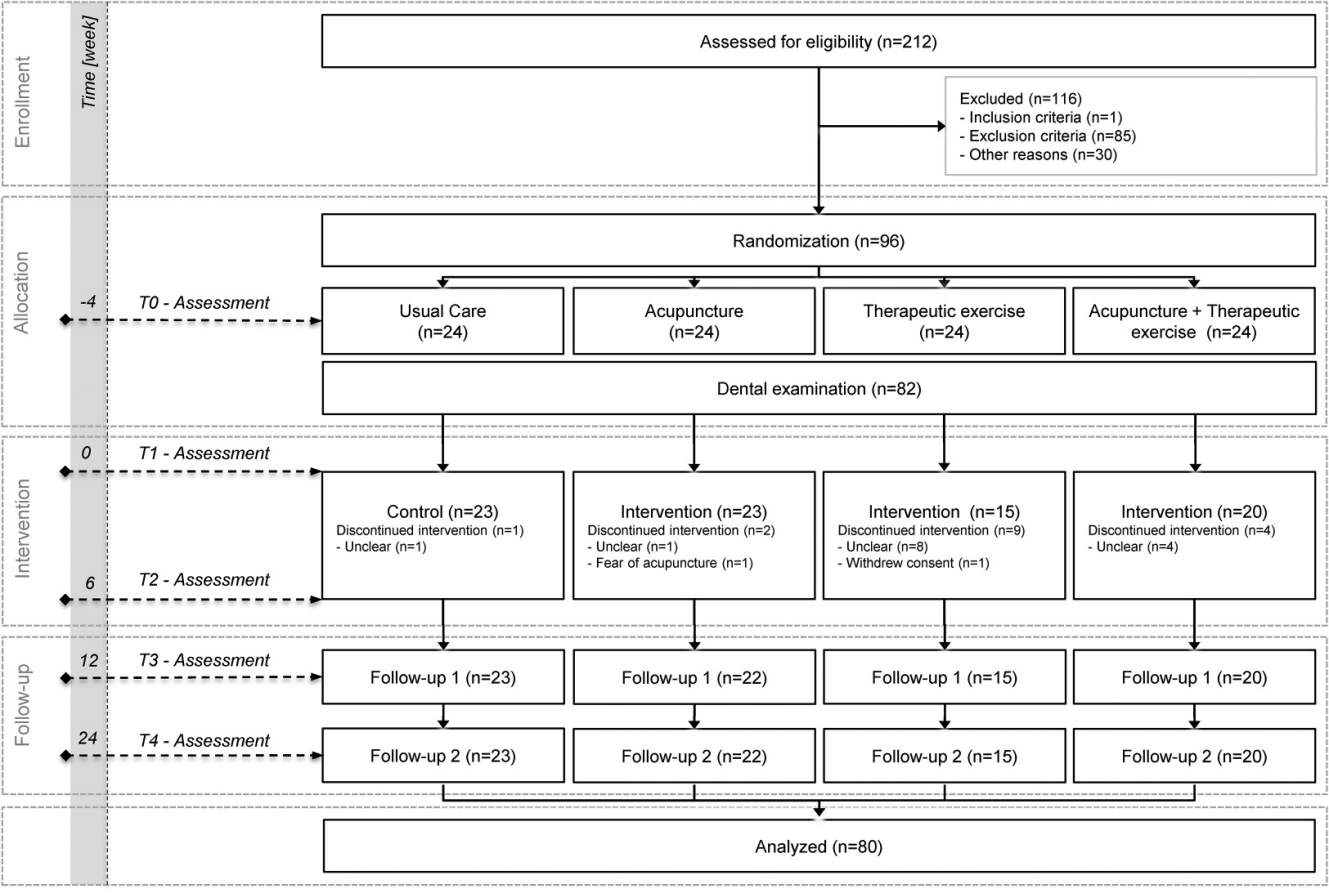
CONSORT flowchart of the study.^13^ *N*, number.

The study flowchart is shown in Figure 1. Of the 96 participants initially included in the study, one participant from the therapeutic exercise group withdrew consent to study participation, so the data from 95 participants were used for the intention-to-treat analysis. No moderate or severe adverse events were recorded during the entire study period.^4^ The basic characteristics of the participants are listed in Table 2. Statistical analysis revealed that apart from the distribution of participants with chronic tension-type headache with pericranial sensitivity (*P = *0.01) the other outcomes and participant characteristics did not differ between the groups (*P *> 0.05).

**Table 2.** Sociodemographic, anthropometric, disease-related, and baseline characteristics of the sample.

Parameter		Total	ANOVA/Chi^2^	Usual care	Acupuncture	Therapeutic exercise	Combination
		Mean (SD)/%	*F*-/Chi^2^- value (*P*-value)	Mean (SD)/numbers	Mean (SD)/numbers	Mean (SD)/numbers	Mean (SD)/numbers
Sociodemographics	Age (years)	38.7 (13.3)	0.18 (0.9)	38.7 (14.6)	39.8 (12.2)	37.0 (15.3)	39.0 (11.6)
	Sex: Female/male (*n*)	75/20	3.5 (0.3)	17/7	18/6	18/5	22/2
ICHD classification	ETTH, no pericranial sensitivity	12	3.0 (0.4)	3	1	3	5
	ETTH, pericranial sensitivity	54	5.1 (0.2)	13	14	17	10
	CTTH, no pericranial sensitivity	5	NA	1	0	3	1
	CTTH, pericranial sensitivity	24	10.7 (0.01)*	7	9	0	8
Pain^13^	Mean intensity (NRS, points)	5.5 (1.2)	0.18 (0.9)	5.5 (1.1)	5.6 (1.3)	5.4 (1.4)	5.5 (1.0)
	Painful days last 3 month (days)	32.0 (27.5)	0.45 (0.7)	36.7 (32.6)	29.6 (24.1)	28.2 (24.5)	33.4 (28.3)
Sum score of the dental examination		4.2 (2.2)	1.1 (0.3)	4.1 (2.5)	4.2 (2.5)	3.6 (1.7)	4.9 (2.2)
Functional Questionnaire Masticatory Organ (temporomandibular dysfunction-score)		20.6 (14.8)	0.57 (0.6)	19.9 (14.1)	21.3 (14.9)	17.8 (16.8)	23.3 (13.6)

All values are separated for group allocation and, likewise, displayed for the total sample. Mean and standard deviations (SDs) are used to describe interval and pseudo-interval values, whereas ordinal and nominal values are displayed as numbers/frequencies. Between group-differences are indicated by the corresponding statistical values for the one way ANOVAs, and Chi^2^-test, respectively. ANOVA: analyses of variances; Combination: acupuncture + therapeutic exercise group; *: statistical significant against the 5% cut-off; NA: not applicable (too low frequencies); ICHD: International Classification of Headache Disorders; TTH: tension-type headache; CTTH: chronic TTH: ETTH: episodic TTH; NRS: numerical rating scale.

**Figure 2.**
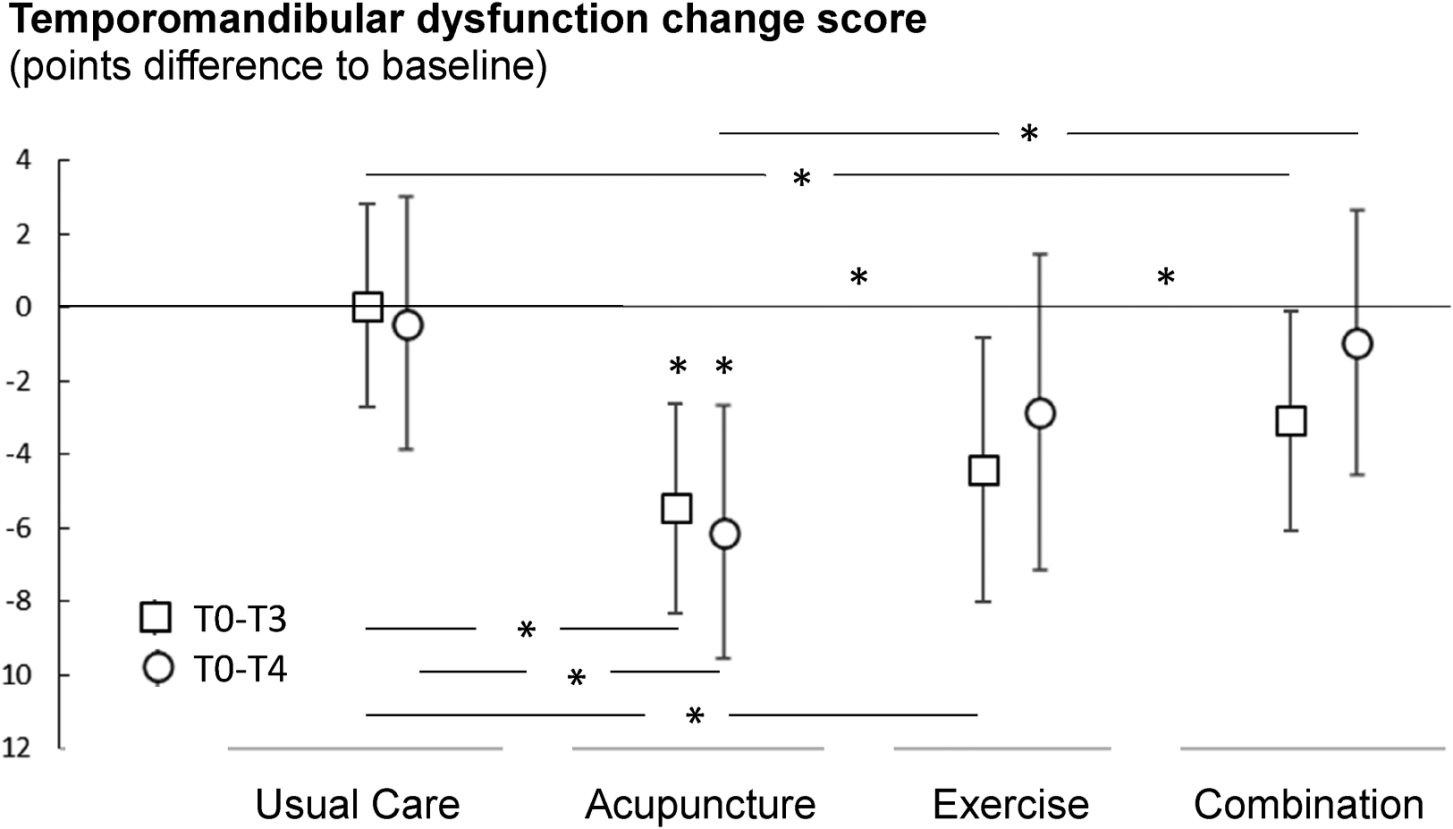
Mean and 95% confidence intervals of the change scores (absolute differences to the baseline assessment) of the temporomandibular dysfunction score at T3 and T4. Values below zero display a decrease in the symptom severity (indicated with * above values), in-line-asterisk indicate between-group-differences.

While the temporomandibular dysfunction score in the usual care group increased by 0.05 points (SD 1.435) between baseline and first follow-up (T3), the values of all intervention groups improved statistically significantly (*F *= 5.1, *P = *0.03). In the acupuncture group, the reduction was −5 (SD 1.436); in the therapeutic exercise group, −4 (SD 1.798) and in the combination group, −3 points (SD 1.504). Six months after the start of the intervention (T4), only the acupuncture group (−6 [SD 1.736]) showed a significant improvement compared to the usual care group (*P < *0.01). There were no significant time×group interactions (*F *= 0.7, *P = *0.5). As a covariate derived from headache, pericranial sensitivity had no influence on the temporomandibular dysfunction-score (*F *= 1.2; *P = *0.3). As a covariate, only the baseline value of the temporomandibular dysfunction score was significant (*F *= 19, *P < *0.001), so the values on which Figure 2 is based were baseline-corrected.

**Figure 3.**
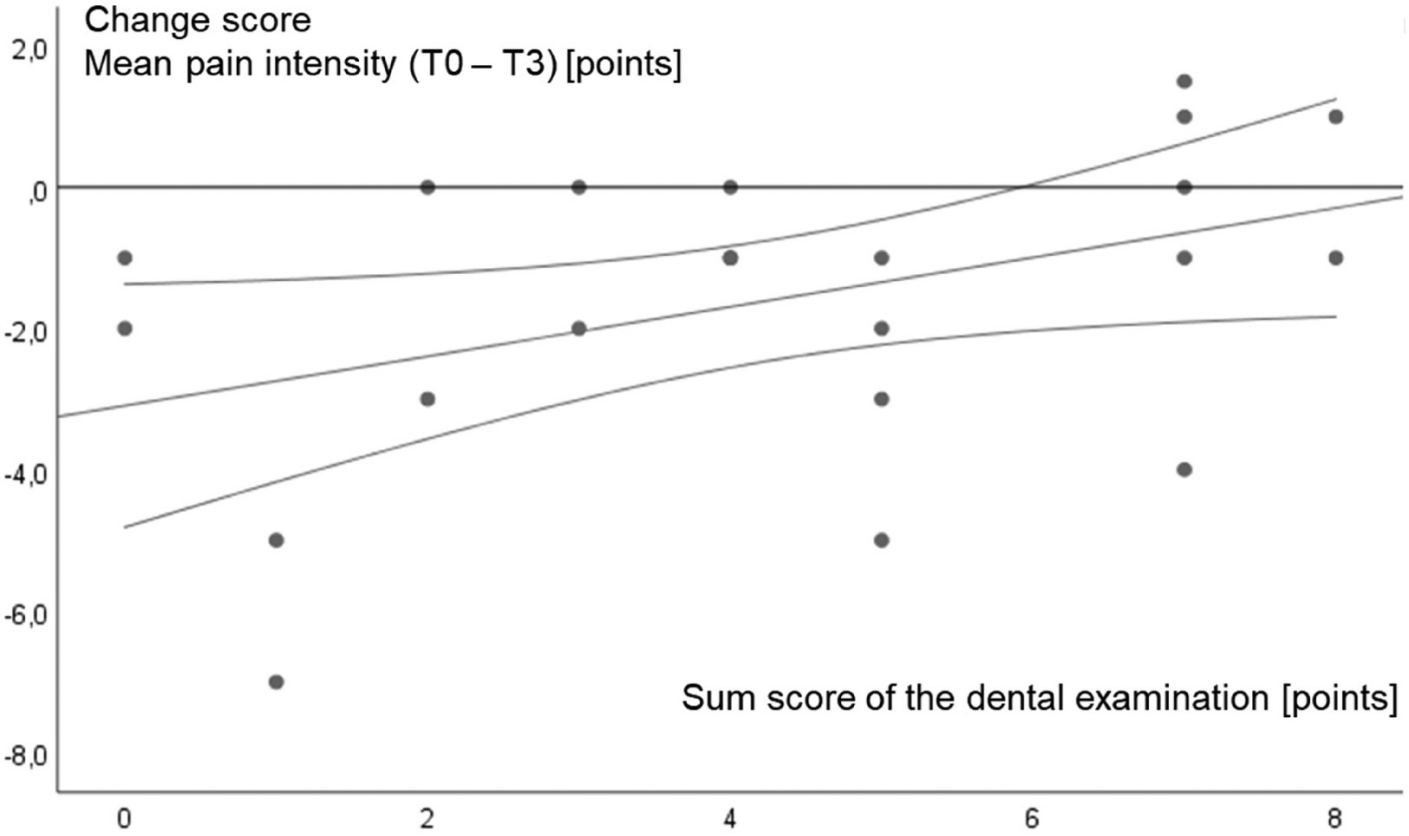
Scatterplot diagram of the sum score of the dental examination versus change score (to T3) of the mean pain intensity correlation in the acupuncture group. The regression curve and its 95% confidence interval are further displayed.

Baseline temporomandibular dysfunction score and dental examination score had no overall effect on change in mean headache pain intensity or frequency of headache days per month. Considering the isolated intervention groups, the dental examination score only affected the change value (up to T3) of the mean (headache) pain intensity in the acupuncture group. The corresponding scatter plot is shown in Figure 3. The 95% confidence interval of the regression curves indicates that participants with a remarkably poor temporomandibular functional status (dental examination score ≥6) can no longer benefit from acupuncture treatment. Tension-type headache therapy with acupuncture is therefore less effective in the case of abnormal dental findings and a resulting high sum score, so successful therapy is more likely in patients with a lower sum score (<6). All other correlation analyses were not significant. All results of the correlation analyses are presented in Table 3.

**Table 3.** Explorative correlation analyses between the sum score of the dental examination and the temporomandibular dysfunction-score at baseline and the interventional effects.

Intervention	Outcome	Mean pain intensity		Days of headache per month	
		Change score to T3	Change score to T4	Change score to T3	Change score to T4
Acupuncture	Temporomandibular dysfunction-score	*R* = −0.287	*R* = 0.063	*R* = −0.147	*R* = −0.261
		*P* = 0.2	*P* = 0.8	*P* = 0.5	*P* = 0.280
	Sum score of the dental examination	*R* = 0.416	*R* = 0.271	*R* = −0.272	*R* = −0.092
		*P* = 0.048*	*P* = 0.2	*P* = 0.2	*P* = 0.7
Therapeutic exercise	Temporomandibular dysfunction-score	*R* = 0.008	*R* = 0.100	*R* = 0.343	*R* = 0.060
		*P* = 1	*P* = 0.7	*P* = 0.3	*P* = 0.9
	Sum score of the dental examination	*R* = 0.190	*R* = −0.062	*R* = −0.252	*R* = 0.045
		*P* = 0.5	*P* = 0.8	*P* = 0.5	*P* = 0.9
Combination	Temporomandibular dysfunction-score	*R* = −0.373	*R* = 0.006	*R* = 0.143	*R* = 0.136
		*P* = 0.1	*P* = 1	*P* = 0.6	*P* = 0.6
	Sum score of the dental examination	*R* = −0.380	*R* = −0.278	*R* = 0.205	*R* = −0.068
		*P* = 0.1	*P* = 0.2	*P* = 0.4	*P* = 0.8

T3: 3 month follow-up; T4: 6 month follow-up; Combination: acupuncture + therapeutic exercise; *: statistical significant against the 5% cut-off.

## Discussion

All interventions targeting tension-type headache produced significant positive effects on the temporomandibular dysfunction score between baseline and 3-month follow-up compared with the usual care group. Sustained significant effects (6-month follow-up) could only be detected after acupuncture. The relevant literature confirms the efficacy of both monotherapies on tension-type headache^2^ and temporomandibular dysfunction.^20^ Temporomandibular joint dysfunction is associated with increased headache intensity and frequency.^21^ It can be assumed that temporomandibular joint dysfunction and headache symptoms influence each other. In contrast to the effects of combination therapy on tension-type headache pain intensity,^4^ no supra-additive effects of combination therapy on temporomandibular dysfunction symptoms could be identified. This may have been caused by the headache-targeted interventions and the increased time required for the combination intervention that could act as a stressor. This may promote the occurrence and sensitization of headache and jaw pain.^22^ In addition, we found that the value of the dental examination score had no effect on headache intensity and frequency when all intervention participants were considered. However, the subsequent group-specific correlation analysis showed that an elevated dental examination score of ≥6 had a negative impact on the effect of treatment using acupuncture on headache intensity. Consequently, the simultaneous presence of severe temporomandibular dysfunction in patients with tension-type headache could explain absent/reduced treatment effects.

Tension-type headache and temporomandibular dysfunction are common coexisting; widespread disorders and the interactions of the two conditions are controversially discussed.^12^ In contrast to the present study, Porporatti et al.^23^ showed that the presence of a primary headache had a negative effect on temporomandibular dysfunction therapy. Cooper and Kleinberg^24^ showed that temporomandibular dysfunction therapy alone led to an improvement of tension-type headache symptoms. Goncalves et al.^25^ treated primary headache and temporomandibular dysfunction simultaneously, thus improving baseline findings on both sides. The results of Carlsson et al.^26^ are in line with the present results, showing that the treatment of tension-type headache alone in the presence of coexisting temporomandibular dysfunction symptoms resulted in an improvement of both syndromes. Improving temporomandibular joint functions by the treatment of tension-type headache is based on central and peripheral pain modulatory mechanisms. The reduction in pain intensity and its frequency have positive effects on temporomandibular joint function due to the same nociceptive system.^11^ Furthermore, the improvement in sleep, the tone-reducing influence on the pericranial muscles and the optimization of the movement behavior of the head/cervical region could be associated with positive functional effects on the temporomandibular function.^26^

A statement of the German Society of Craniomandibular Function and Disorders confirms the effectiveness of physiotherapeutic treatments for temporomandibular dysfunction.^27^ This study also confirmed the medium-term positive effects of physiotherapeutic exercise. In addition to an improvement in blood circulation, regulation of muscle tone, general well-being, and pain perception, positive effects on headache intensity and frequency have been demonstrated.^28^ The results of acupuncture were also comparable with the results of other authors.^29^ The effects of headache treatment on temporomandibular dysfunction symptoms can be explained by the anatomical relationship, the overlapping of peripheral and central nerve structures, and analgesic mechanisms.^30^ Known loco regional effects of acupuncture acting in both tension-type headache and temporomandibular dysfunction include optimization of circulation and muscle tone,^31^ central release of opioid peptides,^32^ and activation of diffuse noxious inhibitory control. The persistent effects of acupuncture could be explained by activation of pain-modulating sensitization systems at the spinal cord level, renewed C-fiber activation, and influences on the hypothalamic and limbic regions.^6^ In addition to these specific mechanisms of treatment effect, non-specific treatment effects play an important role in both acupuncture and therapeutic exercise. However, these effects were held constant by similarly inert therapeutic procedures, comparable expectations, suggestibility, communication, and attention in all groups.

Some limitations were identified during the study process. Eight participants in the medical training group dropped out of the study without giving a reason. This behavior is probably due to the fact that this form of therapy is physically demanding^33^ and is less individualized than acupuncture. In addition, the allocation mode with a presumably increased preference of participants for acupuncture^34^ could explain this absenteeism. Interventions advertised during recruitment may have introduced bias due to particular interest in a treatment modality. In order to assess the influence of these effects, the expectations of the participants were checked and calculated as comparable.^4^ The 60 min exercise duration is quantitatively superior to 30 min of acupuncture and could therefore have an influence on the therapeutic effects. The choice of duration is based on the fact that both therapies correspond to patient expectations and national standards in terms of duration and setting. In addition, it was shown that when comparing the effects of acupuncture with the effects of non-acupuncture, low intensities in the control group led to larger effect sizes for acupuncture.^35^ In order to avoid this distortion caused by too short training times in the therapeutic exercise to counteract this, the duration was set at 60 min. Participants in the usual care group had fewer intervention appointments than participants in the intervention groups. Consequently, this may be responsible for less significant changes due to weaker non-specific therapy effects.^36^ Close monitoring and offering therapeutic exercise or acupuncture treatment after the last follow-up for this group were an attempt to reduce these biases.

Only acupuncture produced a significant and sustained reduction in discomfort in the jaw region. Pronounced dental findings correlating with a high dental examination score negatively influence the efficacy of acupuncture for tension-type headache. Consequently, in treatment-resistant tension-type headache, a dental differential diagnosis based on the present results is recommended to identify barriers to efficacy and to initiate a multidisciplinary therapeutic approach.

**Clinical messages**

- Headache treatment using acupuncture and therapeutic exercise as monotherapy as well as their combination relieves temporomandibular joint complaints.
- Acupuncture has sustained effects on temporomandibular joint complaints.
- Significant dental findings can block the therapeutic effect of headache treatment, so that interdisciplinary diagnosis is recommended in treatment-resistant cases.
